# Typing of HLA susceptibility alleles as complementary tool in diagnosis of controversial cases of pediatric celiac disease

**DOI:** 10.3389/fnut.2025.1500632

**Published:** 2025-02-25

**Authors:** Carolina Naymé Ruera, Luciana Guzman, Lorena Menendez, Laura Orellano, María Cecilia Girard Bosch, Carlo Catassi, Fernando Gabriel Chirdo

**Affiliations:** ^1^Departmento de Ciencias Biologicas, Facultad de Ciencias Exactas, UNLP, Instituto de Estudios Inmunologicos y Fisiopatologicos (IIFP) (UNLP-CONICET), La Plata, Argentina; ^2^Hospital de Niños Superiora Sor María Ludovica, La Plata, Argentina; ^3^Department of Pediatrics, Università Politecnica delle Marche, Ancona, Italy

**Keywords:** celiac disease, HLA, diagnosis, potential CeD, pediatric

## Abstract

**Objectives:**

Diagnosis of celiac disease (CeD), an immune-mediated disorder, is based on clinical presentation, a panel of serological markers, and the histopathological findings in duodenal biopsies. Commonly, pediatric CeD patients fulfill these criteria for diagnosis. However, lack of correlation between serology tests and histology, or no accessible biopsies because of clinical conditions or during the COVID pandemic, are conditions that led to inconclusive diagnoses. Since the majority of CeD patients carry HLA-DQ2 and/or DQ8 alleles, HLA testing is used as a complementary tool in diagnosis though is costly and not broadly available for gastroenterology centers.

**Methods:**

We performed a retrospective study to assess the performance of HLA testing when applied to selected groups of patients who could not be definitely diagnosed following the common algorithm. Eighty patients underwent testing for CeD-related HLA-DQ2 and DQ8 alleles.

**Results:**

HLA typing contributed to diagnosis in 34 patients with positive serology but normal mucosa or those who presented negative serology or slightly positive serology (less than 3 times ULN) and duodenal histopathological changes. In patients with normal histology and negative or slightly positive serology, or those who did not undergo intestinal biopsy (39 in total), HLA typing contributed to CeD diagnosis in 23 cases, only 16 patients were admitted for a clinical follow-up program.

**Conclusion:**

HLA-DQ typing supported the diagnosis in 57 of 80 children (71.2%) with previously inconclusive results, providing a beneficial approach for diagnosing celiac disease (CeD) in selected cases.

## 1 Introduction

Celiac disease (CeD) is one of the most prevalent immune-mediated chronic gut disorders, that develops in genetically susceptible individuals, triggered by the intake of a group of proteins from wheat, barley, and rye, commonly named as gluten. Diagnosis of CeD is based on the clinical presentation, presence of a panel of specific antibodies in peripheral blood, histological demonstration of mucosal damage in duodenal biopsies, and clinical improvement when patient adheres to a gluten-free diet (GFD). CeD may appear at any age with typical gastrointestinal symptoms, but also with minimal and variable intestinal and extraintestinal manifestations, or even with an asymptomatic presentation. CeD is also associated with type I diabetes, autoimmune thyroid disease or other conditions such as Down syndrome and IgA deficiency (1).

Genes encoding for alpha and beta chain of HLA class II molecules are the most strongly genetic factor associated to CeD. Four HLA class II alleles account for the highest relative risk for an HLA-disease association. These are commonly described as HLA-DQ2.5 (encoded by either a *cis* haplotype: HLA-DQA1*0501/HLA-DQB1*0201 or a *trans* haplotype configuration HLA-DQA1*0501/HLA-DQB1*0301 and HLA-DQA1*0201/HLA-DQB1*0202), HLA-DQ2.2 (encoded by HLA-DQA1*0201 and HLA-DQB1*0202), the HLA-DQ8 heterodimer (encoded by the HLA-DQA1*0301 and HLA-DQB1*0302), and rarely the DQ7.5 type (encoded by the HLA-DQA1*05 and HLA-DQB1*0301). Though almost all CeD patients carry one or a combination of these alleles, these are also frequent in non-CeD individuals. Very few CeD cases not carrying any of the HLA susceptibility alleles have been reported (2). Consequently, the absence of these alleles makes very unlikely the disease (high negative predictive value), while their presence is not confirmatory (low positive predictive value) (3, 4).

The prevalence of CeD is estimated at around 1% worldwide, however, this disorder is deeply underdiagnosed. Poor disease awareness, failures in the health system, particularly in developing regions, increasing findings of asymptomatic presentations, and drawbacks during the diagnostic protocol, result in a high rate of undiagnosed patients (5).

Reference centers have followed different algorithms for the diagnosis of CeD, according to their own experiences or performance of each step in the procedure (clinic, laboratory, and pathology evaluations). In the year 2012 the European Society for Pediatric Gastroenterology, Hepatology and Nutrition (ESPGHAN) proposed that the diagnosis of CeD may definitely be established in patients with compatible symptoms having anti-transglutaminase 2 (TG2) IgA levels 10 times above the cut-off value, followed by positive IgA EmA in a second blood sample, and carrying HLA-DQ2 and/or DQ8, avoiding the requirement of the intestinal biopsy (6). More recently, this consensus was reviewed and the current ESPGHAN recommendation considers that positive serology (anti-TG2 IgA levels 10 times above the cut-off value) plus EmA positivity determines the diagnosis even in asymptomatic patients. As HLA typing does not provide a cost-effective outcome, it was not recommended (7).

CeD diagnosis can be reached in most of the cases following these guidelines, however, complex situations are sometimes observed in clinical practice. Asymptomatic presentation, lack of correlation between serology and histological findings, unavailable biopsies, and starting the gluten-free diet (GFD) before performing complete investigations may lead to difficulties in reaching a final diagnosis. Additional studies, such as the evaluation of intraepithelial lymphocytes subsets by flow cytometry (8), biomarkers as intestinal FABP in serum (9, 10), intestinal deposits of anti-TG2 IgA (11), may help to solve these cases, but these tools are still not broadly available.

Algorithms followed by different specialized gastroenterology units may include HLA-DQ typing but this technique is costly and is not easily available for most of the centers (3). Therefore, we aimed to assess the impact of performing HLA-DQ typing when applied to selected groups of patients who could not be diagnosed following the routine protocol as a consequence of normal histology or negative serology, or lack of histopathological assessment due to the lack of endoscopy procedures because of clinical conditions, parental denial, or COVID pandemia.

## 2 Materials and methods

A total of 360 pediatric patients between 1 and 16 years old were diagnosed as CeD in the period between January 2015 and July 2023, at the gastroenterology Unit of Sor María Ludovica Children’s Hospital in La Plata (Argentina). This Hospital is a reference center for the diagnosis of CeD and the patients were referred from different Public Health Units of the Province of Buenos Aires. Patients followed a protocol for Celiac Disease diagnosis in the Gastroenterology Unit.

The diagnosis of CeD was based on the clinical presentation, serology, and histological analysis of intestinal biopsies. Patients with suspected CeD and/or positive serology underwent upper endoscopy, except in those for whom the procedure was not medically indicated. HLA-DQ typing was performed in all patients. After diagnosis, patients were evaluated by a nutritionist to start the GFD. Patients were followed up to monitor dietary compliance to the diet and for clinical examination.

This retrospective study included 80 pediatric patients with suspected CeD whose clinical evaluation, serology, and histopathological findings did not support a definite diagnosis of CeD or those in whom upper endoscopy procedure could not be performed.

Cases diagnosed as CeD on the basis of concordant clinical presentation, positive serology and histopathological findings in the duodenal biopsies, as well as type 1 diabetes mellitus patients, were excluded from this study.

### 2.1 Clinical presentation

Gastrointestinal symptoms included chronic diarrhea, abdominal pain, bloating, and weight loss. Extraintestinal symptoms were anemia, decreased bone mineralization, increased levels of liver enzymes, dermatitis herpetiformis, short stature, and delayed puberty.

### 2.2 Serology

Serum samples were kept frozen at −20°C until analysis in the Immunology Section of the Hospital de Niños Sor María Ludovica. IgA anti-TG2 antibodies were determined by an ELISA test (Quanta Lite R H-tTG ELISA, Inova Diagnostic). IgG anti-DGP were performed by an ELISA test [QUANTA Lite^®^ Gliadin IgG II (DGP), Inova Diagnostics]. Total serum IgA concentration was determined by nephelometric technique (IMMAGE^®^ 800, Beckman Coulter).

Samples presenting IgA anti- TG2 antibodies <20 UA/mL were considered normal. Positive samples were defined as presenting IgA anti-TG2 antibody levels higher than the upper level of normal (ULN). Serology values for IgA anti-TG2 antibodies of 200 UA/mL and 60 UA/mL correspond to 10× ULN and 3× ULN, respectively. All positive samples for anti-TG2 antibodies were evaluated in a second separated blood sample for IgA anti-endomysial antibodies (EMA) (Inova Diagnostics).

### 2.3 Intestinal biopsy

The small intestine biopsy was performed by upper gastrointestinal videoendoscopy under general anesthesia. A Fujinon 530 video esophagoduodenoscope (Video-Gastroscope Fujinon EG-530D) was used. During the procedure, the second portion of the duodenum was reached, 4 samples of the second duodenal portion (D2) and 2 biopsies of the duodenal bulb were taken. The samples were placed in formalin for histological evaluation following Marsh–Oberhuber classification (12).

### 2.4 HLA-DQ typing

Genomic DNA extraction from white blood cells was performed using the QIAamp^®^ DNA Blood Kit (QIAGEN^®^ Inc., Valencia, CA). High resolution HLA genotyping was performed by multiplex polymerase chain reaction (PCR) with biotinylated primers, followed by reverse hybridation of the PCR products with arrays of sequence-specific DQA1 and DQB1 oligonucleotide probes. This is followed by a stringent wash step to remove any mismatched amplified material. Then, streptavidin conjugated with alkaline phosphatase is added and bound to any biotinylated hybrid previously formed; using INNO-LIPA HLA-DQ kits, according to the manufacter’s instructions (Fujirebio, Gent, Belgium). Results were analyzed by using the sixth version of the LiRAS interpretation software for LiPA HLA. The nomenclature was based on “Nomenclature for Factors of the HLA System, 2010” (13).

### 2.5 Patient groups

The patients with suspected symptoms included in the study population were divided into 4 groups as follows:

Group 1: Patients with positive serology and small intestine with normal histology.
Group 2: Patients with negative serology and small intestine with normal histology.
Group 3: Patients with negative serology and small intestine with histopathological changes (Marsh 2 or 3).
Group 4: Patients with who did not undergo intestinal biopsy due to medical contraindication.

When a definite diagnosis could not be reached, patients with symptoms suggestive of celiac disease were included in a follow-up program. In these cases, clinical evaluation was performed every 6 months while serological testing on a normal gluten-containing diet was performed every 12 months.

In the period from 2015 to 2023, patients in Group 1, 2 and 3 underwent endoscopy and histological assessment of intestinal biopsy based on the guidelines from Celiac Disease Expert Committee approved by the National Health Ministry in Argentina (14).

### 2.6 Statistical analysis

Statistical analysis was performed with Graph-Pad Prism software (San Diego, United States). A chi-square test was applied to assess statistical significance.

## 3 Results

In this study, a total of 80 pediatric patients out of 360 with gastrointestinal symptoms did not reach a final diagnosis after the first work-up due to lack of histological evaluation or discrepancy between the serological and histopathological findings. To assess whether HLA-DQ typing can contribute to the diagnosis of CeD, these cases were distributed into the four groups described in section 2.5.

### 3.1 Group 1

**Figure 1:**
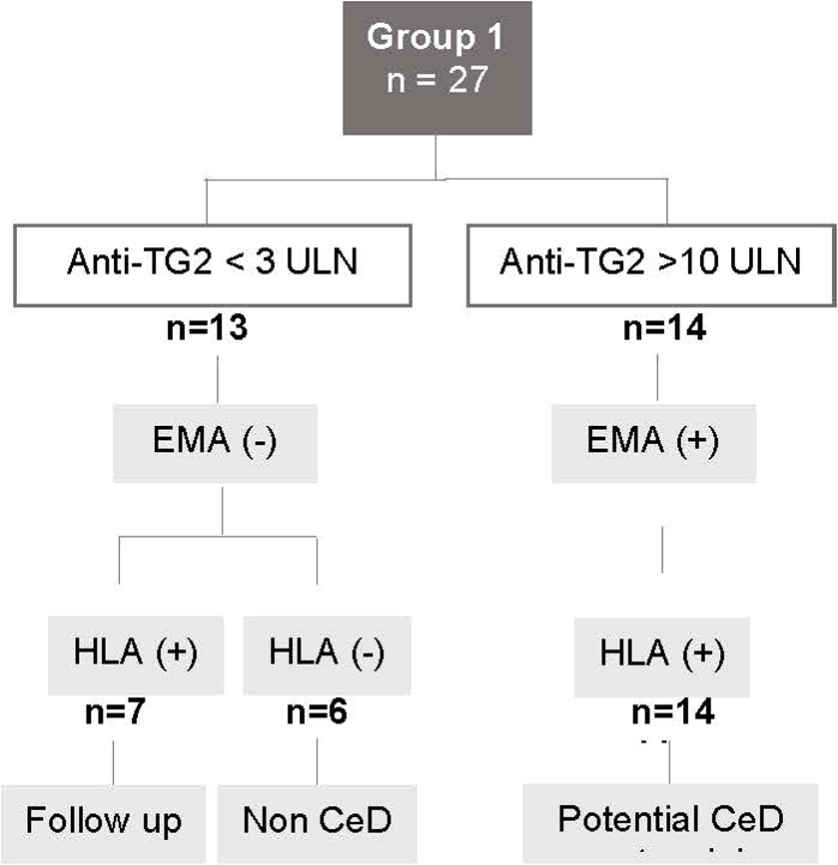
Flowchart for Group 1. Patients with positive serology and small intestine with normal histology. CeD, celiac disease; anti-TG2, anti-transglutaminase 2 antibody levels; ULN, upper limit of normal; EMA, anti-endomysial antibody; HLA, patients carrying the HLA CeD-compatible alleles.

Group 1 included 27 patients with compatible gastrointestinal symptoms and positive serology but a normal villous architecture at the small intestinal biopsy. According to current knowledge, these are cases of “Potential” CeD, a condition that may evolve into fully-expressed CeD or revert to normal over time (5). Fourteen of them (52%) presented both anti-TG2 antibodies at high titer (>10 times ULN) and EMA positivity. All these cases presented compatible HLA and were definitely considered as affected by CeD. Upon discussion with the family, patients started a GFD and all of them showed a positive clinical response (Figure 1). Thirteen other patients had anti-TG2 antibody low levels ranging from 1 to 3 ULN. Among these, six patients were excluded from having CeD as they tested negative for EMA and lacked HLA susceptibility alleles (22%). The remaining seven patients (26%), who had HLA predisposing genes but were EMA negative, were enrolled in a follow-up program and left on a normal diet.

### 3.2 Group 2

**Figure 2:**
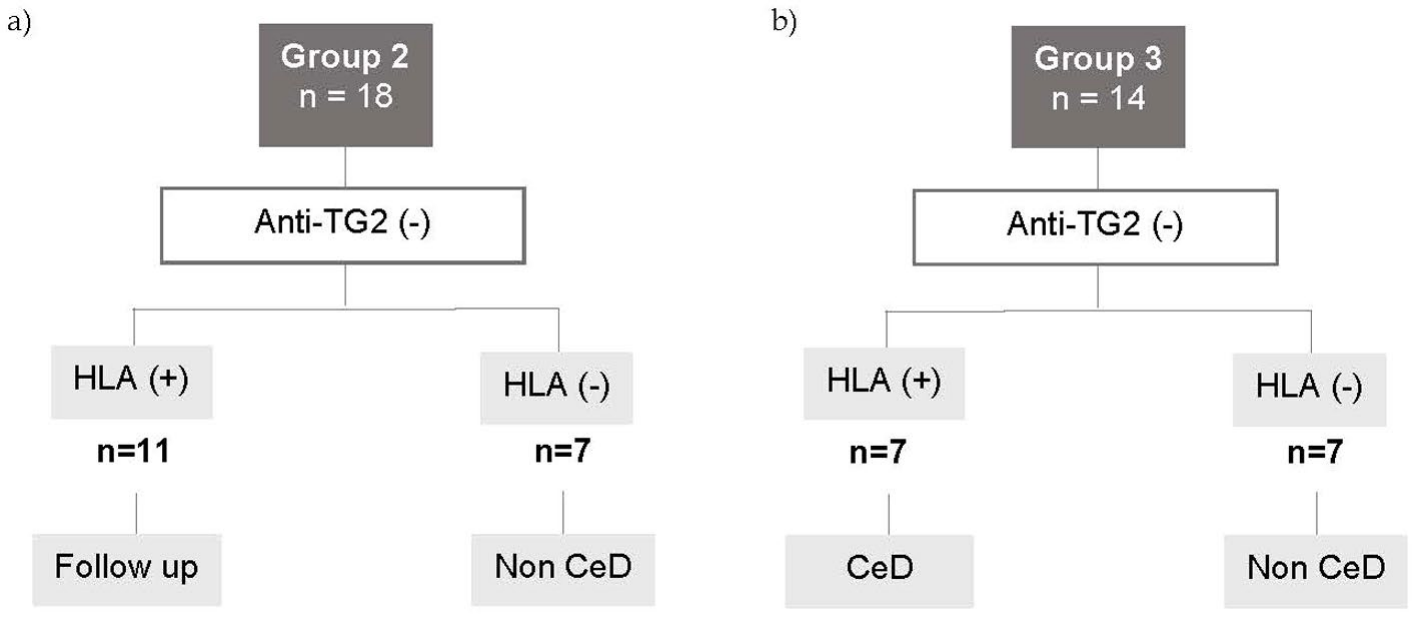
Flowchart for Groups 2 and 3. **(A)** Patients with negative serology and small intestine with normal histology. **(B)** Patients with negative serology and small intestine with histopathological changes. CED, celiac disease; anti-TG2 (−), negative anti-transglutaminase 2 antibody levels; HLA+, patients carrying the HLA CED- compatible alleles.

Group 2 comprised 18 patients who had suggestive symptoms, negative serology, and normal histology. CeD was ruled out in seven patients because they lacked HLA predisposing genes. The remaining 11 patients, with persistence of symptoms and compatible HLA genes, were maintained on a normal diet and entered a follow-up program (Figure 2A).

### 3.3 Group 3

Group 3 included 14 patients with compatible symptoms, negative serology and histopathological changes in the small intestine (Marsh 2, 3 score). HLA predisposing alleles were absent in 7 cases, ruling out CeD. The remaining 7 patients carried HLA susceptibility alleles. Among them, four patients were under 3 years old, exhibited low levels of IgA, and had a Marsh 3 score. A GFD was introduced, leading to a positive clinical response (Figure 2B).

In the search for other causes (i.e., upper digestive bleeding and esophageal atresia), Marsh 3 scores were occasionally observed in the duodenum of two patients. After excluding other conditions such as inflammatory bowel disease, dietary allergies, HIV, and primary immunodeficiency, these two patients were placed on a GFD, resulting in a favorable clinical outcome. Endoscopy and duodenal biopsy 6 months after starting the GFD showed mucosal recovery, and patients were diagnosed with seronegative CeD. The remaining patient presented with severe malnutrition, negative serology, and a Marsh 3 score in the duodenal biopsies. After a positive response to the GFD, and the assessment of duodenal biopsy 6 months later, a diagnosis of CeD was confirmed. The frequency of seronegative CeD was 3/360 CeD patients.

### 3.4 Group 4

Group 4 included 21 symptomatic patients who, due to pre-existing clinical conditions such as heart disease, epilepsy, or severe malnutrition, could not undergo endoscopy. In addition, this group included children who suffered from gastrointestinal symptoms during the COVID-19 pandemic. According to the recommendations of the different Scientific Societies, endoscopy procedures were not performed during this period.

Seven patients presented a negative serology, 4 of them presented a not compatible HLA and CeD was ruled out. The remaining 3 presented compatible HLA and symptoms, therefore were admitted to the follow up program. Eleven cases presented anti-TG2 levels higher than 10 UNL. All of them were EMA positive, carried compatible HLA and were diagnosed as CeD. In these cases, HLA typing was requested following the local guidelines (13).

**Figure 3:**
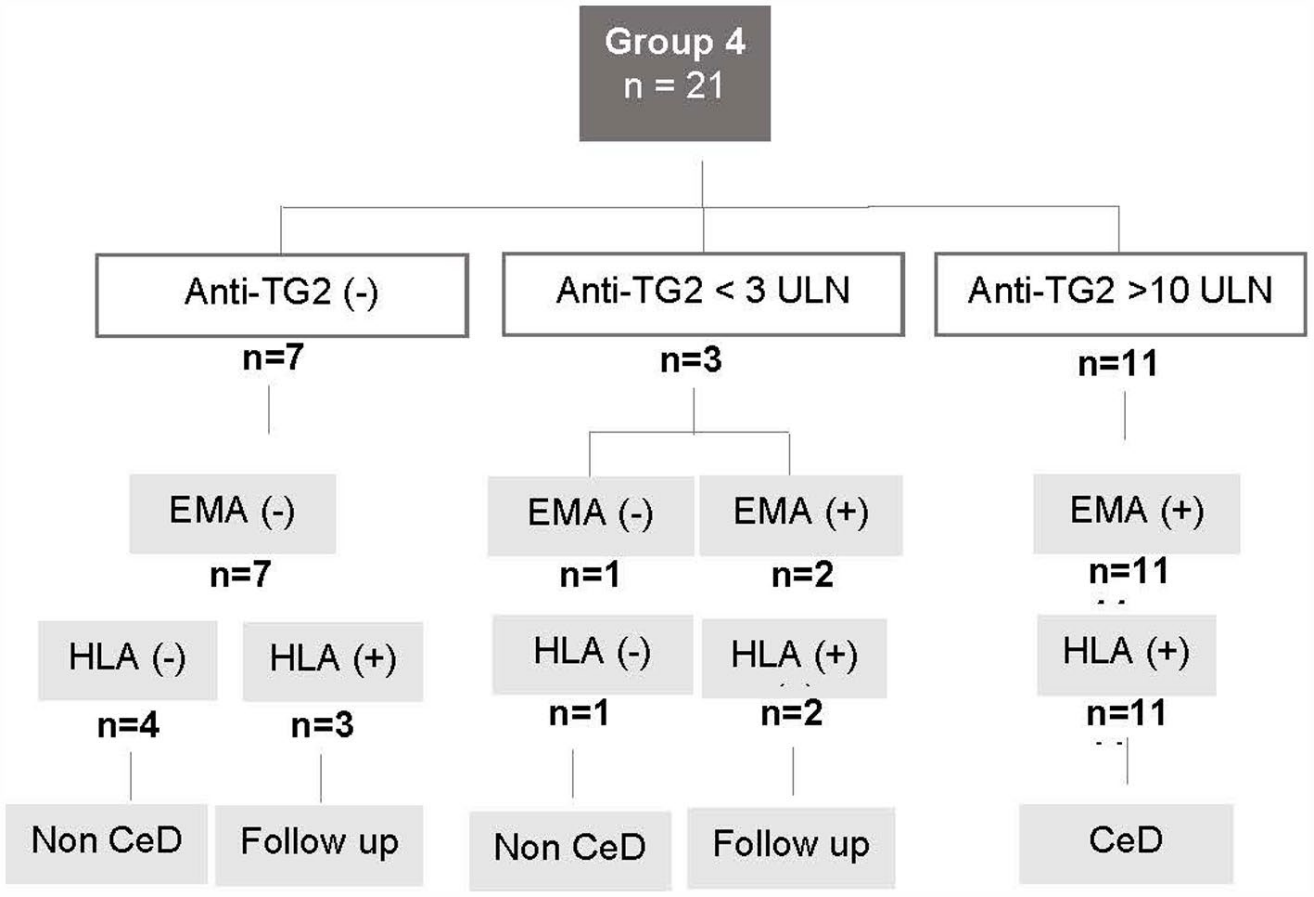
Flowchart for Group 4. Patients with who did not undergo intestinal biopsy due to medical contraindication. CeD, celiac disease; anti-TG2, anti-transglutaminase 2 antibody levels; ULN, upper limit of normal; EMA, anti-endomysial antibody; HLA+, patients carrying the HLA CeD-compatible alleles.

Finally, there were 3 patients with anti-TG2 levels between 1 and 10 UNL and compatible HLA. Two of them were EMA positive and were diagnosed as CeD. Then a good response to the GFD was observed. The remaining case presented anti-TG2 levels below 3 UNL, EMA negative and was admitted in a clinical and serological follow-up program (Figure 3).

## 4 Discussion

Cases showing a discrepancy between the results of serological markers and duodenal histology or those who do not undergo an endoscopy procedure to take duodenal biopsies are common causes for inconclusive CeD diagnosis. HLA typing is a costly technique and is not easily available in many gastroenterology centers. However, this is a valuable tool to exclude the disease when patients do not carry the CeD-compatible alleles and could be beneficial when applied in selected groups of patients.

As the Caucasian central European is the main ethnic contribution to the population studied, DQ2.5 was the most frequent allele found (23 out of 32 diagnosed patients, 16 of them homozygous) (Table 1).

**Table 1:** HLA alleles distribution.

	Group 1 *n* = 27	Group 2 *n* = 18	Group 3 *n* = 14	Group 4 *n* = 21	Total *n* = 80	CeD *n* = 32
DQ2.5/DQ2.5	10	5	1	10	26	16
DQ2.5/x	4	3	2	4	13	7
DQ2.5/DQ8	1	1	—	—	2	1
DQ8/x	3	2	—	2	7	4
DQ2.2/x	—	—	1	—	1	1
DQ7.5	1	—	3	—	4	3
Negative	6	7	7	5	25	—

Studies from European populations have shown the dominance of the DQ2 allele in celiac patients (15). However, other ethnic groups may exhibit different patterns. For instance, research on Indian populations (16) and Native Amerindians in Argentina (17), Chile (18), and Mexico (19) has identified DQ8 as the most frequent allele contributing to CeD. Additionally, the prevalence of the DQ7 allele varies among celiac patients across different communities (20).

**Figure 4:**
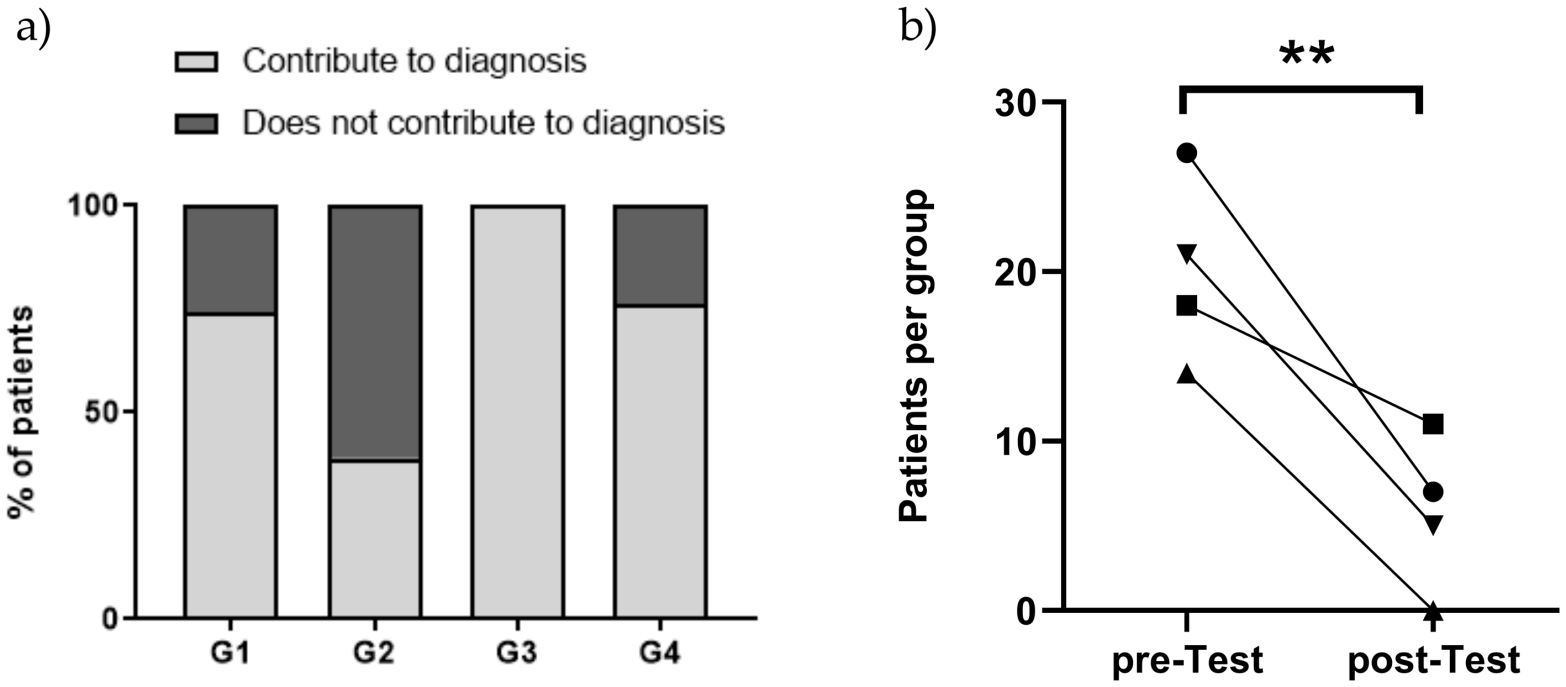
**(A)** Performance of HLA typing to CeD diagnosis per group. **(B)** Impact of HLA typing on diagnosis in study groups. The comparison of diagnosed cases before (pre-test) and after (post-test) HLA typing revealed a significant overall difference (*p* = 0.0016). Statistical analysis for each group is as follows: ● Group 1 (*p* = 0.019), ■ Group 2 (*p* = 0.481), ▲ Group 3 (*p* = 0.0001), ▼ Group 4 (*p* = 0.027). A chi-square test was applied to assess statistical significance.

Altogether, this study shows that HLA typing may contribute as a useful complementary tool in the diagnosis of CeD (Figure 4A). Group 1 included patients with so-called potential CeD (5), a situation that may be difficult to ascertain in clinical practice due to the discrepancy between the results of CeD serology and intestinal histology. HLA determination was a useful confirmatory test in 52% of these cases, but at the same time helped to exclude CeD in further 22%, therefore leaving only 26% of patients in need of follow-up to reach a final diagnosis. In Group 2, cases presented the typical condition in which absence of HLA susceptibility alleles ruled out the pathology, while the low level of antibodies or negative serology determined that patient entry in a follow-up program. In Group 3, all cases received a conclusive diagnosis either because they were CeD or disease was excluded. Noteworthy in this group, two patients younger than 3 years old presented with severe malnutrition, abdominal distention, and chronic diarrhea. Serology was negative, likely secondary to their poor nutritional state. Histopathological assessment revealed severe villous atrophy. These patients followed a GFD with good response. In these cases, HLA evaluation supported the diagnosis. HLA typing was found helpful to select a group of seronegative or presenting low anti-TG2 antibody levels individuals at risk (17), or to exclude the disease when HLA was not compatible. Group 4 included patients evaluated during the COVID pandemia. As a referral center in the Buenos Aires Province, 400 endoscopies are performed in average, with 40–50 diagnosis of CeD per year. However, during the pandemia, endoscopy procedures were solely performed in extreme emergency cases. As a result, HLA evaluation was useful in supporting the CeD diagnosis in these cases. Considering the results across all study groups, it is evident that HLA typing contributed in making a diagnostic decision in a significant number of cases (*p* = 0.0016) (Figure 4B). As mentioned before, the highest contributions were observed in Groups 1, 3, and 4, where HLA typing contributed to the diagnose in 74, 100, and 76% of cases, respectively. In Group 2, HLA typing contributed to the diagnosis in only 39% of cases.

As indicated, patients with persistent and isolated low level of anti-TG2 antibodies were included in a clinical and serological follow up protocol. Some of these patients will be eventually diagnosed as CeD, while others may normalize the serology. In a prospective study, Auricchio et al. (21) reported that 32% of 280 potential CeD patients enrolled in the study have presented negative serology in successive blood samples and none of them developed villous atrophy over 60 months of follow up. This exemplifies the complex scenario for CeD diagnosis for some of the cases. Although not tested in our study due to the limited number of cases, DQ typing may also be beneficial for individuals already on a gluten-free diet (GFD) (22). This aspect is highly relevant and warrants further evaluation in referral centers that could enroll large number of patients.

HLA typing has typically been used as an exclusion criterion in the absence of susceptibility HLA. However, various diagnostic algorithms have highlighted the importance of positive results as well. The study by Lionetti et al. (23) demonstrated the value of HLA typing as a mass screening tool in the pediatric population.

## 5 Conclusion

In summary, though HLA-DQ typing is not required for CeD diagnosis in all cases, it is a valuable complementary tool in evaluating cases with suspected CeD, emphasizing its crucial role in ruling out the disease when negative and providing diagnostic support in challenging clinical situations. Since it is expensive and not broadly available, here we show that its use in selected groups of patients such as in the context of serology-histology discrepancy, lack of upper endoscopy and histological assessment, contribute to the diagnosis of celiac disease.

## Data Availability

The raw data supporting the conclusions of this article will be made available by the authors, without undue reservation. In adherence to ethical guidelines, all patient data were anonymized to protect confidentiality.
